# Formal consensus method for the development of a primary outcome measure to assess the clinical and cost-effectiveness of cardiac magnetic resonance imaging after primary percutaneous coronary intervention pathway activation (PIPA feasibility study)

**DOI:** 10.1186/1745-6215-14-S1-P48

**Published:** 2013-11-29

**Authors:** Maria Pufulete, Chiara Bucciarelli-Ducci, Rachel Brierley, Chris Rogers, Jessica Harris, Barney Reeves

**Affiliations:** 1 University of Bristol, Bristol, UK

## Background

PIPA is designed to determine the feasibility of a prospective, multi-centre cohort study to assess the clinical and cost-effectiveness of cardiac magnetic resonance imaging (CMR) in patients who activate the primary percutaneous coronary intervention (PPCI) pathway. A key PIPA objective is to define a primary composite outcome that represents a clinically important change in management (proxy outcome for reduction in the future risk of a major adverse cardiovascular event (MACE)) as a result of an eligible patient having had CMR. A formal consensus method based on the modified nominal group technique[1] is being used to define this outcome.

## Methods

Formal consensus is being developed in three phases: (1) Systematic review to identify relevant literature; (2) Formulation of statements about how CMR changes patient management; (3) Consensus process, in which an interdisciplinary panel of stakeholders will complete an online survey (rating statements for importance on a 9-point Likert scale), attend a face-to-face consensus meeting to discuss variation in anonymised survey responses, then re-rate the survey statements.

## Results

Sixteen statements will be rated by twelve panellists (interventional cardiologists, CMR experts, cardiac network directors, and GPs). Final survey results will be presented as medians, interquartile ranges (IQR), counts and percentages. Statements with median scores of 7–9 and IQR of <3 will be considered to be in consensus and used to define the primary outcome.

## Conclusions

The primary composite outcome will be used to evaluate the effectiveness and cost-effectiveness of CMR to change clinical management in ways expected to prevent future MACE in this population.
